# After 40 Years, Retina Reveals It Uses Positive Feedback, as Well as Negative

**DOI:** 10.1371/journal.pbio.1001058

**Published:** 2011-05-03

**Authors:** Richard Robinson

**Affiliations:** Freelance Science Writer, Sherborn, Massachusetts, United States of America

**Figure pbio-1001058-g001:**
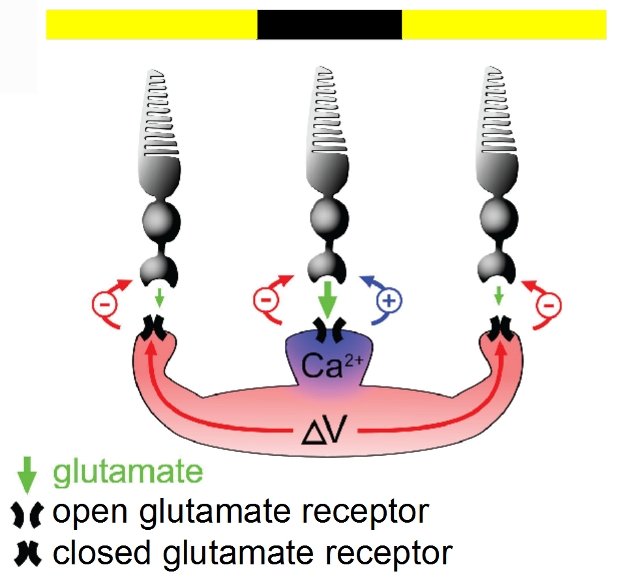
Horizontal cells in the retina have a positive feedback synapse onto photoreceptor cells that locally offsets a previously known negative feedback connection.

[Fig pbio-1001058-g001]The eye is not a camera, and the retina is not a piece of film. Indeed, the retina might be better likened to a computer running Photoshop, given the extent of image processing that it performs before passing visual information along to the brain. A central aspect of that processing is called center-surround inhibition, in which illumination stimulates the firing of a small number of retinal cells, accompanied by inhibition of surrounding cells. This phenomenon increases spatial contrast and sharpens perception of edges.

The mechanism of center-surround inhibition is complex, but one key part is played by interactions between photoreceptor cells and horizontal cells. Photoreceptors—the rods and cones that absorb light to begin the visual process—form synapses with horizontal cells, which, as their name implies, spread out horizontally across the retinal surface. Each horizontal cell receives input from dozens of photoreceptor cells, integrating and processing their input before passing it further along the visual chain.

While photoreceptor cells activate horizontal cells, horizontal cells also subsequently influence photoreceptor cells, inhibiting their activation, an inverse influence called a negative feedback loop. But in this issue of *PLoS Biology*, Skyler Jackman, Richard Kramer, and colleagues show that the two are also linked by a positive feedback loop, which may help offset the loss of signal strength inherent in negative feedback, helping to maintain strong signal output without degrading spatial contrast.

The authors began by isolating whole retinas from the anole lizard. Unlike most other sensory cells, photoreceptors continually release neurotransmitter in the *un*stimulated condition, that is, in the dark. The neurotransmitter glutamate is released from small sacs called synaptic vesicles that fuse with photoreceptor membrane. After their contents are released, bits of the membrane pinch off to generate new vesicles that gradually refill with glutamate for another round of release. The authors tapped into this recycling process to stain the vesicles with a dye as they were forming, allowing them later to monitor the rate of exocytosis of the labeled vesicles, and thus, the degree of stimulation of the photoreceptor.

Because the negative feedback effect of horizontal cells activated by glutamate is to inhibit photoreceptor exocytosis, the authors expected that addition of exogenous glutamate into the synapse would reduce the rate of exocytosis. Instead, they found the opposite—an increase in glutamate release with addition of glutamate, the essence of a positive feedback loop.

Through a series of experiments that blocked or stimulated different receptors, they established that the effect was mediated by a certain kind of receptor, called an ionotropic glutamate receptor. Since such receptors are found on the surface of the horizontal cells, but not on photoreceptor cells, and since ablating horizontal cells eliminated the effect, the authors concluded that positive feedback originated in these horizontal-cell receptors. Activation of the receptor allows calcium ions to flow into the horizontal cells, and when the authors released calcium directly into the horizontal cells, the photoreceptors were stimulated even in absence of excess glutamate, clinching the case. Further experiments will be needed to identify the messenger released by the horizontal cell that stimulates the photoreceptor cell. More experiments will also be needed to confirm these results in rods, since the anole lizard's retina contains only cones.

How has this positive feedback mechanism remained hidden in plain sight through four decades or research on the retina? One reason, the authors suggest, is that it is only robustly observed in whole retina preparations, while retinal slices have been used for the majority of work elucidating retinal function.

The authors propose that the function of the positive feedback loop is to offset the effect of negative feedback, which reduces overall output from a given visual stimulus. By tightly controlling the spatial spread of the positive stimulatory signal, they suggest that the system can maintain high signal strength without losing the sharp contrast enhancement of the center-surround system.

**Jackman SL, Babai N, Chambers JJ, Thoreson WB, Kramer RH (2011) A Positive Feedback Synapse from Retinal Horizontal Cells to Cone Photoreceptors. doi:10.1371/journal.pbio.1001057**

